# Supplementary material for: Content-rich biological network constructed by mining PubMed abstracts
Source: BMC Bioinformatics. 2004 Oct 8;5:147. doi: 10.1186/1471-2105-5-147 (PMC528731; doi:10.1186/1471-2105-5-147)
Supplement: Additional File 5 — The original Chilibot query results of the term "long-term potentiation (LTP)" and 22 other terms, limiting the latest references analyzed to the years 1990, 1995, 2000, and 2004. [file 1471-2105-5-147-S5.bz2 › chilibotAdditionalFile5/ltp2004/html/PKA.html]

 


**PKA** (Input: PKA ) 

---


**Google Searches:** Entire Web  | EDU domain only  | PDF files only

.

**External Links:** OMIM |
LocusLink
| Swissprot
| GeneCards

  
**Maps of PKA**

Simple
Complete
graph in
radiant
tree
square
layout.

**New Hypothesis !**

**Synonyms** 

- pka   [PubMed]

**Synopsis**

- Our data show that genetic inhibition of **PKA** impairs L LTP by reducing **PKA** mediated suppression of PP1 2A.  Eur J Neurosci, 2002 
  [25]

- Preliminary results suggest that D1 receptor stimulation or **PKA** activation leads to increased surface GluR1 expression in PFC neurons  Ann N Y Acad Sci, 2003 
  [23]

- These results suggest that calcineurin activation accompanied by **PKA** inhibition in a PN causes dephosphorylation of DARPP 32, which releases PP 1 from inhibition.  J Neurosci, 2002 
  [23]

- These results suggest that activation of CaMKII, **PKA** and PKC may be crucial for the induction and the early phase  J NeurophysiolJ Neurophysiol, 2002 
  [23]

- Our results suggest that activation of presynaptic **PKA** can enhance neurotransmission at corticostriatal synapses.  Neurosci Lett, 2002 
  [23]

- These results suggest that activity dependent mechanisms operate through an AC1 **PKA** signaling pathway to target some synapses for consolidation and others for elimination during barrel map formation.  Nat Neurosci, 2003 
  [23]

- These results suggest that NO, cGMP, and PKG cause release of calcium from ryanodine sensitive stores, which in turn causes phosphorylation of CREB in parallel with **PKA** during the induction of L LTP.  J Neurophysiol, 2002 
  [20]

- Recently, we found that a repeated activation of protein kinase A **PKA**,   Neurosci Res, 2003 
  [19]

- Our findings suggest that **PKA** mediated phosphorylation of the active zone protein RIM1alpha at a single N terminal site induces presynaptic LTP.  Cell, 2003 
  [19]

- Previous studies documented an integral role for the cAMP **PKA** system in late LTP and recently, inhibition of cAMP degradation facilitates LTP and ameliorates mnemonic deficits.  Neuroscience, 2003 
  [19]

- The new synapses are enriched in NMDA receptor and it was found that their generation involves activation of NMDA receptors, **PKA** and CREB.  Neurol Neurochir Pol, 2003 
  [18]

- These results support the point that the **PKA** signaling pathway is crucial for neuronal plasticity in visual cortex, and the dissociation of the role of **PKA** and PKG in long term synaptic plasticity in the visual cortex suggests that LTP alone is NOT sufficient to support ocular dominance plasticity, or LTD plays a more fundamental role than LTP in ocular dominance plasticity.  Cereb Cortex, 2003 
  [17]

- Activation of apostsynaptic **PKA** is necessary for the expression of LTP in CA1 pyramidal neurons and is sufficient for initiating persistent synaptic facilitation.  J Neurosci, 2003 
  [17]

- The released NE and dopamine exert their role in modulating synaptic plasticity via activation of beta adrenergic and D1 D5 receptors, respectively, which in turn increase the levels of cytoplasm adenosine cyclic monophosphate and **PKA**.  Eur J Neurosci, 2002 
  [16]

- Previousreports show that bath application of the adenosine 3 5 cyclic monophosphate cAMP analog, Sp cAMPS, induces a protein kinase A **PKA** dependent and protein synthesis dependent long term potentiation  [LTP]  LTP at hippocampal CA3 CA1 synapses.  Synapse, 2003 
  [16]
